# Editorial: ASICs: structure, function, and pharmacology, part II

**DOI:** 10.3389/fphys.2024.1390239

**Published:** 2024-03-08

**Authors:** Enrique Soto, Candice Askwith

**Affiliations:** ^1^ Instituto de Fisiología, Benemérita Universidad Autónoma de Puebla, Puebla, Mexico; ^2^ Department of Neuroscience, The Ohio State University, Columbus, United States

**Keywords:** ASIC, pain, drug addiction, pH, pharmacology, cardiovascular, protons, channels

In the period from 2022 to 2024 by using a search in PubMed for “Acid sensing ion channels ASIC” either in the title, the abstract or the key words, within the years 2022–2023 retrieved 75 documents. Which compared to the previous annual trend of publication suggest that the field has a declining rate of publication (MacLean and Soto, 2022). Yet, critical insights are still being made and advancing our understanding of the roles of acid sensing ion channels.

Significant progress has been made in elucidating the structure, function, and pharmacology of ASICs. Replacing the structural representations of ASICs as static, depicted in manuscripts as crystalline structures by the truly dynamic nature of ASIC molecules. Rather than rigid structures, ASICs exhibit dynamic movements, including the rotation of subunits, the bending of channel regions, and the displacement of specific structural components that contribute to their functional versatility.

Advancements in understanding ASIC dynamics have revealed crucial insights into their function. This includes the characterization of open-close gate states, the complex process of desensitization, and interactions with the surrounding membrane and ions in both intra and extracellular environments. Additionally, studies have shed light on the role of scaffolding elements in channel trafficking and membrane localization. These provide a more realistic understanding of ASICs and ionic channels in general, moving beyond traditional schematics (Kasimova et al., 2020; Klipp and Bankston, 2022; Cullinan et al., 2023).

ASICS have long been known to participate in sensory transduction and have been linked to temperature, touch and pain sensing (Price et al., 2000; Price et al., 2001; Gregory et al., 2018). Recent work indicates that ASIC1 is critical for tactile discrimination by responding to protons released by Merkel cells in response to mechanical stimuli (Yamada et al., 2024). These results suggest that proton-mediated signaling may be an important component of sensory neuron function. The study by Tkachenko et al. discovered that an increase in temperature enhances the nociceptive responses of C-fibers to acidic pH, ATP, and high K^+^ concentration. To investigate the temperature dependence of nociceptive C-type dorsal afferent neurons to mechanical and thermal stimuli, the researchers utilized an *ex vivo* mouse hind limb skin-saphenous nerve preparation. Action potentials from the saphenous nerve were extracellularly recorded. The basal discharge rate and conduction velocity of nociceptive fibers were studied.

The authors examined the temperature dependence of nociceptor responses to high potassium concentration, ATP, and acidic pH at three different temperatures: 20°C, 30°C, and 40°C. All examined fibers were sensitive to high potassium concentration solution perfusion in their receptive field regardless of the temperature of the solution. Only approximately half of the fibers were sensitive to ATP at 20°C, but increasing the temperature to 40°C resulted in 100% of the fibers being sensitive. Notably, at 20°C, all observed fibers were unresponsive to acidic pH, but at 40°C, this number increased to nearly 90%. The results indicate that temperature changes within physiological limits for the skin constitute a significant variable that modulates the excitability of nociceptive fibers and their response to endogenous signaling molecules.

The role of Acid-Sensing Ion Channels (ASICs) in drug abuse-related synaptic changes has been a significant focus of research in the field. In the study by Gupta et al., the mechanisms underlying the effects of ASICs on synaptic plasticity and drug-induced effects in medium spiny neurons of the nucleus accumbens in mice were investigated. This study raises the possibility that manipulating ASIC1A therapeutically may counteract drug-induced synaptic changes and associated behaviors.

It is well-established that drug abuse induces synaptic transmission changes in the reward mesolimbic circuit, which are believed to underlie drug craving and relapse. ASIC1A subunits are notably expressed in the nucleus accumbens, particularly in medium spiny neurons expressing D1 and D2 receptors (Kreple et al., 2014a). Disruption or knockout of ASIC1A in mice leads to synaptic rearrangements in the nucleus accumbens that resemble those observed during cocaine withdrawal.

Additionally, Gupta et al. shown that ASIC1A null mice exhibit increased cocaine-conditioned place preference, dendritic spine density, and increased expression of AMPA receptors compared to NMDA receptors. The authors demonstrated that the increased recruitment of GluA2-lacking calcium-permeable AMPA receptors contributes to the elevated AMPA/NMDA receptor expression ratio in null mice. These findings show that insights into the effects of ASICs on synaptic plasticity and drug-induced effects highlight the therapeutic potential of manipulating ASIC1A in the treatment of cocaine addiction.

The commentary by Bader et al. on the Gupta et al. manuscript emphasizes the significance of the results and underscores the need for further investigation into the relationship between ASIC activation and dopamine D1 receptors. The authors underline the potential role of ASIC1A in drug-related addiction, spanning various substances such as opioids and alcohol. This commentary underscores the importance of ASIC1A as a crucial area for future research and study in understanding addiction mechanisms (Harmata et al., 2023).

In the pursuit of new selective modulators for ASICs, Evlanenkov et al. highlight the absence of modulators targeting ASIC2. Their study focuses on the effects of four derivatives of 2-aminobenzimidazole, a known modulator of ASIC3 currents, on ASIC ionic currents in rat brain neurons and in a heterologous expression system in CHO cells.

The authors discovered that a compound called Ru-1355 strongly enhanced responses of ASIC2a and moderately potentiated native ASICs as well as heteromeric ASIC1a/ASIC2a channels. Conversely, the most active compound, Ru-1199, exhibited lower selectivity for ASIC2, also potentiating ASIC1a and native ASICs. Other compounds, such as Ru-1270 and Ru-1199, did not produce significant modifications in ASIC currents.

Mechanistic analysis suggests that the compounds potentiate a specific open state with slow kinetics, which is weakly sensitive to the ASIC pore blocker diminazene. These findings shed light on potential avenues for developing selective modulators targeting ASICs, particularly ASIC2, with implications for understanding their physiological roles and therapeutic applications.

The role of ASICs in various pathological processes represents a significant advancement in our understanding of their function. ASICs have been implicated in nociception and pain, migraine, and even the invasive capacity of cancer cells. In this Research Topic a review authored by López-Ramírez and González-Garrido, delves into the role of ASICs in cardiovascular function.

Authors discuss the distribution and potential functions of ASICs, highlighting that she ASIC1, ASIC2a, and ASIC3 subunits are expressed in the myocardial cell membrane, with ASIC1 and ASIC2a also found within the cell nucleus. The localization of ASIC1 and ASIC2a subunits within the nucleus of cardiomyocytes is intriguing, and their functional role remains to be fully elucidated. Additionally, ASIC2-containing channels are expressed in nodose ganglion terminals innervating the aorta, where they participate in the baroreceptor reflex (Lu et al., 2009). ASIC3-containing channels are expressed in dorsal root ganglion neurons (DRG) nociceptor neurons innervating the heart, mediating the pain sensation associated with ischemic events that result in an excess of lactic acid in the extracellular environment and subsequent ASIC3 activation (Immke and McCleskey, 2001).

The authors conclude that a comprehensive analysis of the role of ASIC channels in the cardiovascular system is critical. Additionally, further investigation into the mechanisms regulating cardiovascular function is necessary to explore the potential of ASIC-targeted therapies. This underscores the importance of ongoing research into ASICs and their potential as therapeutic targets for cardiovascular conditions.

Significant advances in our understanding of ASICs have indeed been made, particularly regarding their role in drug abuse, pharmacology, and pathology. Furthermore, there has been notable progress in understanding the modulation of ASICs by neuropeptides. However, as highlighted by Evlanenkov et al., there is still a need for more selective molecules that can serve as pharmacological tools to study the role of ASICs in both physiology and pathology.

It is expected that this Research Topic of papers will contribute to furthering the progress of ASIC research and enhancing our understanding of the specific roles of ASICs in physiology, pathology, and their pharmaceutical potential. Overall, continued research in this area is crucial for unlocking the full potential of ASICs as therapeutic targets and their diverse roles in various physiological and pathological processes.
